# Atherosclerotic Plaque Fissuration and Clinical Outcomes in Pre-Diabetics vs. Normoglycemics Patients Affected by Asymptomatic Significant Carotid Artery Stenosis at 2 Years of Follow-Up: Role of microRNAs Modulation: The ATIMIR Study

**DOI:** 10.3390/biomedicines9040401

**Published:** 2021-04-08

**Authors:** Celestino Sardu, Pietro Modugno, Gaetano Castellano, Lucia Scisciola, Michelangela Barbieri, Lella Petrella, Mara Fanelli, Gabriella Macchia, Eugenio Caradonna, Massimo Massetti, Giuseppe Paolisso, Raffaele Marfella

**Affiliations:** 1 Department of Advanced Medical and Surgical Sciences, University of Campania “Luigi Vanvitelli”, 80100 Naples, Italy; lucia.scisciola@unicampania.it (L.S.); michelangela.barbieri@unicampania.it (M.B.); giuseppe.paolisso@unicampania.it (G.P.); raffaele.marfella@unicampania.it (R.M.); 2 Department of Cardiovascular Sciences, Gemelli Molise S.p.a, 86100 Campobasso, Italy; pietro.modugno@gemellimolise.it (P.M.); gaetano.castellano@gemellimolise.it (G.C.); eugenio.caradonna@gemellimolise.it (E.C.); massettimas@yahoo.it (M.M.); 3 Department of Molecular Diagnostic, Gemelli Molise S.p.a, 86100 Campobasso, Italy; lella.petrella@gemellimolise.it (L.P.); mara.fanelli@gemellimolise.it (M.F.); gabriella.macchia@gemellimolise.it (G.M.); 4 Department of Cardiovascular Sciences, IRCCS A. Gemelli University Policlinic Foundation, 00168 Rome, Italy; 5 Mediterranea Cardiocentro, 80122 Naples, Italy

**Keywords:** atherosclerotic plaque, pre-diabetes, inflammation, microRNAs, metformin therapy

## Abstract

Atherosclerotic plaque instability and rupture in patients with asymptomatic carotid artery stenosis (ACAS) is a leading cause of major adverse cardiac events (MACE). This could be mainly evidenced in patients with pre-diabetes. Indeed, the altered glucose homeostasis and insulin resistance could cause over-inflammation of atherosclerotic plaque, favoring its conversion to unstable phenotype with rupture and MACE. Notably, metformin therapy reducing the metabolic distress and the inflammatory burden could reduce MACE in ACAS patients with pre-diabetes. In this setting, the microRNAs (miRs) could be used as molecular biomarkers of atherosclerosis progression, plaque rupture, and worse prognosis in normoglycemics (NG) versus pre-diabetics metformin users (PDMU) versus pre-diabetics non-metformin users (PDNMU). However, our study aimed to investigate a wide miRNA panel in peripheral blood exosomes from patients with ACAS divided in NG versus PDMU versus PDNMU, and to associate the circulating miRNA expression profiles with MACE at 2 years of follow-up after endarterectomy. The study included 234 patients with ACAS divided into NG (*n* = 125), PDNMU (*n* = 73), and PDMU (*n* = 36). The miRs’ expression profiles of circulating exosomes were determined at baseline and at 2 years of follow-up by Affymetrix microarrays from the patients’ plasma samples from any study cohort. Then we collected and analyzed MACE at 2 years of follow-up in NG versus PDMU versus PDNMU. Prediabetics versus NG had over-inflammation (*p* < 0.05) and over expressed miR-24 and miR-27 at baseline. At 2 years of follow-up, PDNMU versus NG, PDMU versus NG, and PDNMU versus PDMU over-expressed inflammatory markers and miR-24, miR-27, miR-100, miR-126, and miR-133 (*p* < 0.05). Finally, at the end of follow-up, we observed a significant difference about MACE comparing PDNMU versus NG (*n* = 27 (36.9%) versus *n* = 8 (6.4%); *p* < 0.05), PDNMU versus PDMU (*n* = 27 (36.9%) versus *n* = 6 (16.6%); *p* < 0.05); and PDMU versus NG (*n* = 6 (16.6%) versus *n* = 8 (6.4%); *p* < 0.05). Admission glucose values (HR (hazard ratio) 1.020, CI (confidence of interval) 95% (1.001–1.038), *p* = 0.029), atheromatous carotid plaque (HR 5.373, CI 95% (1.251–11.079), *p* = 0.024), and miR-24 (HR 3.842, CI 95% (1.768–19.222), *p* = 0.011) predicted MACE at 2 years of follow-up. Specific circulating miRs could be over-expressed in pre-diabetics and specifically in PDNMU versus PDMU after endarterectomy. MiR24, hyperglycemia, and atheromatous plaque could predict MACE at 2 years of follow-up.

## 1. Background

The rupture of atherosclerotic plaque is a leading cause of major adverse cardiac events (MACE) in patients with asymptomatic carotid artery stenosis (ACAS) [1]. Indeed, the significant atherosclerotic plaque narrowing and the conversion of the atherosclerotic plaque from a stable to an unstable phenotype could both contribute to the plaque ulceration and rupture [1,2]. Then, the atherosclerotic plaque ulceration and rupture could cause the MACE [3]. Notably, the shifting from stable to unstable plaque phenotype with its consequent rupture could involve multiple processes via the over inflammation [1,2,3]. To date, in these patients, the endarterectomy is a recommended treatment to remove the atherosclerotic plaque and to ameliorate the clinical outcomes [4]. On the other hand, a higher percentage of asymptomatic patients, and particularly those at high risk as the patients with diabetes mellitus, could experience a worse prognosis despite endarterectomy [4]. Indeed, in diabetics versus normoglycemics, the altered glucose homeostasis and the insulin resistance could cause the over-expression of inflammatory molecules at the level of atherosclerotic plaque, leading to unstable plaques rupture and the MACE [4]. Intriguingly, similar mechanisms have been reported for patients with pre-diabetes [5]. Indeed, the pre-diabetes is an intermedia clinical condition between normoglycemia (NG) and the diabetes mellitus, characterized by altered glucose homeostasis, insulin resistance, and over-inflammation [5]. Intriguingly, in pre-diabetics, the amelioration of glucose homeostasis and insulin resistance induced by hypoglycemic drugs as the metformin could reduce the inflammatory burden [5,6]. This could then result in the control of atherosclerotic processes [5,6]. At the clinical level, all these effects metformin-induced could reduce the MACE in patients with pre-diabetes [6]. On the other hand, there are no conclusive data about the molecular (anti-inflammatory), metabolic (amelioration of glucose homeostasis and insulin resistance), and clinical effects (rate of MACE) induced by metformin in pre-diabetics with ACAS after carotid endarterectomy. Moreover, the identification of serum biomarkers could be crucial to predicting plaque rupture and MACE in prediabetics versus NG, and specifically in prediabetics under metformin therapy (PDMU) versus pre-diabetics without metformin therapy (PDNMU). To date, few microRNAs (miRs) have been recently evaluated in this respect [7]. The miRs are small, noncoding, regulatory RNAs composed of 18 to 22 nucleotides that regulate target gene expression at the post-transcriptional level by either inhibiting translation or causing degradation of the corresponding messenger RNA [7]. Moreover, the miRs have been evaluated in the progression and rupture of unstable carotid artery plaques in ACAS patients [7,8]. In detail, the authors showed that the miR-24, miR-27, miR-100, miR-126, and miR-133 are involved in the pathological processes, then leading to the atherosclerotic plaque instability of carotid vessels [7,8]. Notably, these pathological processes were linked to the different miRs’ expression, and evidenced in patients with fissuration and rupture of the unstable carotid plaques [7,8]. Therefore, from here we might speculate that miR-24, miR-27, miR-100, miR-126, and miR-133 could be implied in fissuration and rupture of the unstable plaques for ACAS patients with pre-diabetes versus normoglycemics.

Therefore, our study hypothesis was that metformin therapy, reducing inflammatory burden, could modulate miRs’ expression in ACAS patients, leading to a reduction of MACE after endarterectomy. Moreover, in the present study, we evaluated the inflammatory burden and circulating miRs in NG versus PDNMU versus PDMU at baseline and at 2 years of follow-up after carotid endarterectomy. Finally, for these three cohorts of patients, we evaluated the rate of MACE at 2 years of follow-up after carotid endarterectomy.

## 2. Research Design and Methods

In an observational, multicenter study we investigated consecutive inpatients with ACAS of both sexes undergoing carotid endarterectomy as recommended by international guidelines [9]. Thus, the ACAS patients undergoing carotid endarterectomy evidenced stenosis of the carotid artery >70% [9]. To evaluate the entity of carotid atherosclerotic plaque-related stenosis and to provide information on the composition of the plaque, we used the duplex ultrasound and the computed tomographic angiography [9]. The degree of carotid stenosis was determined according to the NASCET (North American symptomatic carotid endarterectomy trial) criteria [10]. All the enrolled patients received an optimal medical treatment to limit disease progression via the control of atherosclerosis risk factors as smoking, hypertension, diabetes mellitus, and dyslipidemia, with antiplatelet agents, statins, and angiotensin-converting enzyme inhibitors [10]. The ACAS patients underwent a baseline clinical examination, gave their medical history, and had never developed neurologic symptoms or cerebral lesions assessed by computed tomography [10]. Finally, all the recruited patients before the intervention received computed tomography or magnetic resonance imaging to evaluate the carotid stenosis [10].

Then, from the study population we identified the NG patients and those with pre-diabetes. The pre-diabetes was categorized according to the American Association of Clinical Endocrinologists’ criteria and the American Diabetes Association [11]. Furthermore, the patients with pre-diabetes answered a specific questionnaire about the use of metformin before the beginning of the study, the dates of the beginning and the end of treatment, the route of administration, and the duration of use [11].

To date, we classified the PDMU patients as pre-diabetics who used metformin therapy for at least 6 months. The PDNMU were the pre-diabetics that did never assume metformin and/or that assumed metformin for a period <6 months. The study population was recruited from the Department of Cardiology and Cardiovascular Surgery of the Gemelli Molise, Campobasso, and from the Department of Advanced Medical and Surgical Sciences, University of Campania “Luigi Vanvitelli”, Naples, Italy, from January 2016 to June 2018. In the overall population and in three cohorts of study (NG, PDNMU, PDMU), authors performed the carotid sonography by a single ultrasound machine (Toshiba Medical Systems Co, Ltd., Tokyo, Japan). The study was approved by the local Ethics Committee (number 440) and informed written consent was obtained for each patient. The study was performed in accordance with the Declaration of Helsinki.

The study endpoints were evaluated in the 3 study cohorts (NG, PDNMU, PDMU) after endarterectomy at 2 years of follow-up. The enrolled patients respected the following inclusion and exclusion criteria.

### 2.1. Study Inclusion and Exclusion Criteria

The inclusion criteria were: patients aged >18 and <75 years with indication to receive a carotid endarterectomy for extracranial high-grade (>70%) internal carotid artery stenosis; patients without a confirmed diagnosis of diabetes mellitus; patients without chronic inflammatory disease; patients without the neoplastic disease.

Exclusion criteria were: diagnosis of diabetes mellitus; patients with clinical or laboratory evidence of heart failure; patients with the previous endarterectomy for significant carotid stenosis; patients with the previous stroke, valvular heart defects, malignant neoplasms, or secondary causes of hypertension.

### 2.2. Laboratory Analysis

In the overall study population, authors measured, after an overnight fast, the plasma glucose, HbA1c (glycated hemoglobin A1c), and serum lipid levels by enzymatic assays in the hospital chemistry laboratory. However, the levels of fasting blood glucose were evaluated before surgery. Fasting and postprandial plasma glucose data were obtained from the average of each assessment. From peripheral blood samples at baseline and at follow-up end, we evaluated inflammatory markers as C reactive protein (CRP), interleukine 6 (IL6), and tumor necrosis factor alpha (TNFα). At baseline and at follow-up end, we evaluated the miRs implied in atherosclerotic carotid plaque instability and rupture for ACAS patients as miR-24, miR-27, miR-100, miR-126, and miR-133 [8,9].

### 2.3. MicroRNAs Isolation and Reverse-Transcription Real Time PCR (qRT-PCR)

MiRs were extracted from blood sample using the miRNeasy Kit (Qiagen; Hilden, Germany) according to the manufacturer’s instructions. Total RNA, including miRNAs, was extracted using Trizol Reagent (Invitrogen, Carlsbad, CA, USA) reagents according to the manufacturer’s instructions. The RNA yield and concentrations were determined by Bioanalyzer Agilent 2100 (Duarte, CA, USA), and all RNA samples were stored at −80 °C until use. MiRs’ expression was analyzed using the two-step protocol of TaqMan MicroRNA Assays (Applied Biosystems, Foster City, CA, USA), according to the manufacturer’s instructions. Briefly, 5–10 ng of total RNA were reverse transcribed using miRNA-specific primers for hsa miR-24 3p (Applied Biosystems, Foster City, CA, USA), hsa miR-27b-3p (Applied Biosystems, Foster City, CA, USA), hsa miR-100-5p (Applied Biosystems, Foster City, CA, USA), hsa miR-126-5p (Applied Biosystems, Foster City, CA, USA), hsa miR-133a-3p (Applied Biosystems, Foster City, CA, USA), and hsa miR-133b (Applied Biosystems, Foster City, CA, USA). The hsa miR-638 (Applied Biosystems, Foster City, CA, USA) was used to normalize miRs expression combined with reagents (dNTPs, reverse transcription buffer, RNase inhibitor, reverse transcriptase, and H2O nuclease free) from the Taqman miRNA Reverse Transcription kit (Applied Biosystems, Foster City, CA, USA). The reactions were performed for 30 min at 16 °C, 30 min at 42 °C, 5 min at 85 °C, and then stored at 4 °C. Real-Time quantitative Polymerase Chain Reaction (RT-qPCR) was subsequently conducted using specific TaqMan probes of the TaqMan microRNA Assay kits (Applied Biosystems, Foster City, CA, USA) to quantify miRNA expression. The reactions were incubated in a 96-well plate at the following thermal cycling conditions: 2 min at 50 °C, 20 s at 95 °C, (3 s at 95 °C and 30 s at 60 °C) for 40 cycles. Data analysis Relative quantification of miRNA expression was calculated with the 2- ΔΔCt method [9]. Therefore, all data were normalized using the endogenous miR-638 expression, as previously reported in a similar model of miRs’ analysis in ACAS patients [8]. Indeed, according to the signal data from the microarrays, we used miR-638 as the reference miR for normalization because it showed the most homogeneous expression levels, in terms of coefficient of variance, in our samples (mean probe signal: 7.4; SD (standard deviation): 0.3; CV (coefficient of variance): 3.7%). Finally, only the data with a Ct value <30 for miR-638 were included in the evaluation.

### 2.4. Carotid Ultrasound

Two experienced physicians in sonography, blinded to study protocol, performed the carotid ultrasound exam for every enrolled patient. However, for all the study population, a high-resolution B-Mode, color Doppler, and pulse Doppler ultrasonography of extracranial arteries was performed at admission to the hospital (before intervention) and at 2 years of follow-up with the ultrasound machine Toshiba Aplio Power Vision (Toshiba Medical Systems Co, Ltd., Tokyo, Japan) equipped with 4–11 MHz linear array transducer. The procedures were all standardized to avoid bias between operators, and the patients were examined in a supine position with the head tilted backward [9,10]. Then, we classified the ultrasonography findings for each carotid as normal, stenosis <50%, stenosis 50% to 70%, stenosis >70%, and as total occlusion [10]. Then, we examined the plaque morphology and the atherosclerotic lesions by their echogenicity as echolucent (soft, lipid rich), moderately echogenic (heterogeneous or fibrotic), and dominantly echogenic (calcified) [10]. Finally, operators examined the surface structure of atherosclerotic plaque to individuate the presence of ulcerations or thrombus and then to define the plaques as ulcerative or smooth and thrombotic, respectively [10]. Briefly, the significant carotid stenosis (stenosis > 70%) was diagnosed by the measurement of an increase in the peak systolic velocity (PSV) > 2.1 m/s and the end-diastolic velocity (EDV) > 0.7 m/s [9,10]. Finally, the degree of carotid stenosis was also assessed by measuring the vessel diameter at the point of maximal stenosis compared with the plaque-free vessel diameter distally from the lesion [9,10]. The follow-up of patients included clinical visits and carotid ultrasound at 2 years of follow-up, to detect possible vascular events and to assess stenosis progression, respectively.

### 2.5. Intervention: Endarterectomy and Carotid Revascularization

After the ultrasonography the ACAS patients received the carotid endarterectomy. This intervention was made at the operator’s discretion, preferentially using the eversion technique with peri-procedural shunt whenever feasible [9]. The treated patients underwent intervention under general anesthesia, and monitored by transcranial Doppler ultrasonography (TDU) and electroencephalographic (EEG) registration. However, operators used a shunt on the basis of EEG and ICD (international diagnostic codex) criteria [9]. Before cross-clamping, an intravenous bolus of heparin (5000 IU) was administered, and all endarterectomies were open with careful dissection of the bifurcation into the internal and external carotid arteries [9]. The operators then used venous patches in case of indicated patch closure [9]. To date, they used a Dacron patch only in case of insufficient venous material. After the intervention, the plaques were categorized into 3 groups based on their overall appearance: fibrous, fibro-atheromatous, or atheromatous [10]. In addition, in peri-procedural medical management, all patients received optimal medical treatment and antiplatelet therapy as recommended by international guidelines [10].

### 2.6. Clinical Visits, Data Collection and Analysis

Physicians evaluated at baseline and at follow-up the clinical characteristics of the study population as NG versus PDNMU versus PDMU. The data were collected and analyzed during clinical visits 10 days after clinical discharge, and at 2 years of follow-up by the treating physician, by telephonic interviews, hospital admissions, and discharge schedules [10]. Therefore, at the follow-up visits, the physicians blinded to study protocol evaluated the clinical status of each patient and performed a physical examination with the collection of vital signs and of adverse events [10]. Thus, physicians evaluated the adherence to drug therapy and any clinical symptom referred by any patient [10]. Therefore, the authors evaluated specifically the MACE at follow-up end, collecting the data prospectively from electronic medical records used in the clinical setting at participants’ Institutions. However, the authors used the electronic systems for data capture, collection, and monitoring, with on-site and real timing data entry. To date, the authors collected the patients’ files in each participating Institution that were then analyzed.

### 2.7. Major Adverse Cardiac Events Definition

Authors evaluated the MACE at 2 years of follow-up in the three cohorts of study as the PDNMU, the PDMU, and the NG. The authors defined the MACE as a composite endpoint indicating cardiovascular disease events, hospital admissions for heart failure, and ischemic cardiovascular events. The cardiovascular disease events were diagnosed by evidence of ischemic heart disease, peripheral arterial disease, stroke/transitory ischemic attack, or revascularization procedure [10,12]. Finally, the study investigators reported all the events with the potential to be adjudicated as one of the predefined study endpoints, regardless of the opinion of the investigator [12]. In the case of identification of a suspected unreported event by a reviewer, the authors asked the reviewer to make a note back to the investigator [12]. Thereafter, authors collected the MACE during patients’ interview, visits, and by hospital discharge schedules [12].

### 2.8. Study Endpoints

The research was conducted in the overall population and selectively in the three different cohorts, as NG, PDNMU, and PDMU. However, in the NG, PDNMU, and PDMU patients, the authors evaluated the different expression of circulating inflammatory molecules (CRP, IL-6, and TNFα), and of circulating miRs (miR-24, miR-27, miR-100, miR-126, and miR-133) implied in inflammatory alterations of atherosclerotic carotid plaque, and plaque instability [7,8]. The inflammatory molecules and miRs were evaluated at baseline before the intervention and at 2 years of follow-up after endarterectomy. Finally, in the three different cohorts, we evaluated the rate of MACE at 2 years of follow-up.

### 2.9. Statistical Analysis

A qualified physician with expertise in statistics performed all the statistical analyses by SPSS version 23.0 (IBM statistics, Chicago, IL, USA). Therefore, the categorical variables were presented as frequencies (percentages), and continuous variables as mean ± SD. Then, the Chi-squared analysis or Fisher’s-exact test were used to compare categorical data between groups. The independent samples Student t-test or ANOVA test was used to compare normally-distributed continuous data between groups, and the Mann-Whitney U test was used to compare the distribution of continuous skewed data between groups. Correlation performed using Pearson’s correlation analysis and Spearman’s correlation analysis in the case of skewed variables. The MACE rates were derived as Kaplan-Meier estimates and compared by log-rank test. Multivariate Cox regression analysis was performed for estimating the relationships among study variables (age, BMI (body mass index), hypertension etc.) and MACE at 2 years of follow-up. Overall survival and event-free survival were assessed by Kaplan-Meier survival curves and compared by the log-rank test. The resulting hazard ratios (HRs) and 95% Cis (confidence of intervals) were reported. Two-tailed *p* values < 0.05 were considered statistically significant.

## 3. Results

The overall population was comprised of 234 patients with ACAS, divided into NG (*n* = 125), PDNMU (*n* = 73), and PDMU (*n* = 36). At baseline, NG versus PDNMU, and versus PDMU presented a significantly lower rate of hypertension, hypercholesterolemia, ischemic heart disease, BMI, systolic blood pressure, and diastolic blood pressure (*p* < 0.05). See Table 1. Notably, PDNMU versus NG, and PDNMU versus PDMU had higher values of HbA1C, glucose, and of the Homeostatic Model Assessment of Insulin Resistance (HOMA-IR), as for total cholesterol, HDL (high density lipoprotein)/LDL (low density lipoprotein) cholesterol, and triglycerides (*p* < 0.05). See Table 1. Regarding atherosclerotic plaque morphology, we found a higher rate of Atheromatous plaque in PDNMU versus NG (*n* = 36 (49.3) versus *n* = 45 (36); *p* < 0.05), and in PDMU versus NG patients (*n* = 16 (44.4) versus *n* = 45 (36); *p* < 0.05). See Table 1. Finally, a significantly higher percentage of PDNMU versus NG and of PDMU versus NG were under anti-hypertensive medications as ACEi, ARBs, diuretics, and calcium blockers, and under statins’ therapy (*p* < 0.05). See Table 1.

Regarding circulating miRs’ at baseline, we found a higher expression of miR-24 comparing PDNMU versus NG, PDNMU versus PDMU, and PDMU versus NG (*p* < 0.05). See Table 2. This trend was confirmed for miR-27, comparing PDNMU versus NG, PDNMU versus PDMU, and PDMU versus NG (*p* < 0.05). See Table 2. At baseline, PDNMU versus NG, as well as PDNMU versus PDMU, over-expressed miR-100 (*p* < 0.05). See Table 2.

Regarding circulating miRs’ at 2 years of follow-up after endarterectomy, we found an over-expression of miR-24, miR-27, miR-100, miR-126, and miR-133 in PDNMU versus NG, PDMU versus NG, and PDNMU versus PDMU (*p* < 0.05). See Table 2.

Notably, we found a significant down regulation of miR-24 *p* < 0.05), miR-27 (NG: 0.13 ± 0.02 versus 0.91 ± 0.04 arbitrary unit (A.U.); PDNMU: 0.95 ± 0.10 versus 2.06 ± 0.21 A.U.; PDMU: 0.58 ± 0.09 versus 1.54 ± 0.30 A.U.; *p* < 0.05), miR-100 (NG: 0.028 ± 0.0004 versus 0.77 ± 0.04 A.U.; PDNMU: 0.089 ± 0.006 versus 1.76 ± 0.22 A.U.; PDMU: 0.059 ± 0.001 versus 0.80 ± 0.12 A.U.; *p* < 0.05), miR-126 (NG: 2.79 ± 1.98 versus 45.64 ± 23.49 A.U.; PDNMU: 29.55 ± 5.89 versus 108.65 ± 42.71 A.U.; PDMU: 10.43 ± 5.84 versus 134.22 ± 70.58 A.U.; *p* < 0.05), and miR-133 (NG: 0.30 ± 0.06 versus 10.82 ± 1.44 A.U.; PDNMU: 1.74 ± 0.28 versus 14.96 ± 2.22 A.U.; PDMU: 0.94 ± 0.28 versus 9.14 ± 2.16 A.U.; *p* < 0.05), comparing, respectively, their values at 2 years of follow-up versus baseline expression for each cohort of study.

Regarding inflammatory markers, PDNMU versus NG, and PDMU versus NG over-expressed at baseline CRP, IL6, and TNFα (*p* < 0.05). See Table 2. At 2 years of follow-up, PDNMU versus NG, PDMU versus NG, and PDNMU versus PDMU over-expressed CRP, IL6, and TNFα (*p* < 0.05). See Table 2.

Finally, at follow-up end, we observed a significant difference about MACE (*n* = 41 (17.5%)), comparing PDNMU versus NG (*n* = 27 (36.9%) versus *n* = 8 (6.4%); *p* < 0.05), PDNMU versus PDMU (*n* = 27 (36.9%) versus *n* = 6 (16.6%); *p* < 0.05), and PDMU versus NG (*n* = 6 (16.6%) versus *n* = 8 (6.4%); *p* < 0.05). See Table 2.

From the Cox regression analysis, admission glucose values (HR 1.020, CI 95% (1.001–1.038), *p* = 0.029), atheromatous carotid plaque (HR 5.373, CI 95% (1.251–11.079), *p* = 0.024), and miR-24 (HR 3.842, CI 95% (1.768–19.222), *p* = 0.011) predicted MACE at 2 years of follow-up. See Table 3.

**Figure 1:**
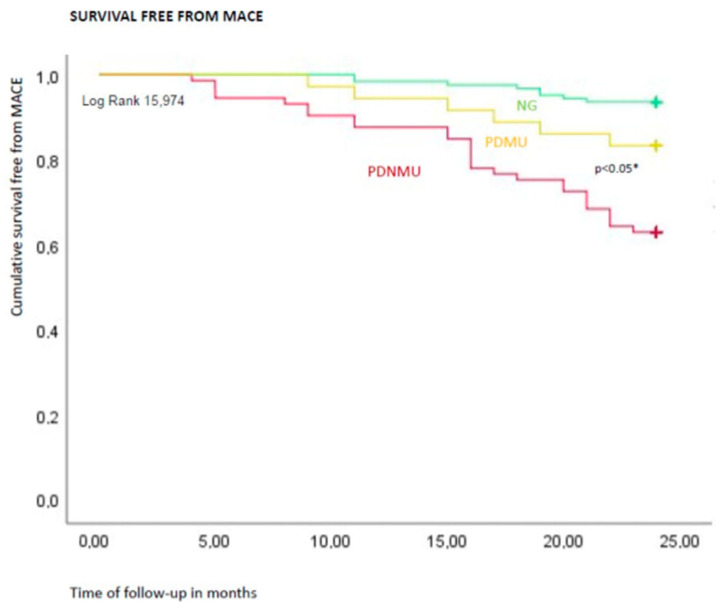
The Kaplan curves for freedom from Major Adverse Cardiac Events (MACE) at 2 years of follow-up in study cohorts. In this figure, the Kaplan curves for freedom from MACE at 2 years of follow-up (24 months) comparing pre-diabetics non-metformin users (PDNMU) versus normoglycemics (NG), pre-diabetics metformin users (PDMU) versus NG, and PDNMU versus PDMU. The curves showed a significant difference regarding freedom from MACE at 2 years of follow-up (24 months) comparing PDNMU versus NG, PDMU versus NG, and PDNMU versus PDMU (*p* < 0.05). The NG are represented in green color, the PDMU in yellow, and the PDNMU in red color. * Is for statistical significant (*p* < 0.05).

The Kaplan curve showed the significant difference regarding freedom from MACE at 2 years of follow-up (24 months) comparing PDNMU versus NG, PDMU versus NG, and PDNMU versus PDMU (*p* < 0.05). See Figure 1.

## 4. Discussion

For the first time in literature, we evidenced in our study in ACAS patients with pre-diabetes versus normoglycemics the baseline over-expression of inflammatory molecules and circulating miR-24 and miR-27. Notably, the PDNMU versus PDMU overexpressed circulating miR-100, while, at 2 years of follow-up, the PDNMU versus the NG, the PDMU versus the NG and the PDNMU versus the PDMU overexpressed the inflammatory markers, and the circulating miR-24, miR-27, miR-100, miR-126, and miR-133. On the other hand, at 2 years of follow-up after endarterectomy, all the study cohorts evidenced a significant miRs’ downregulation. Notably, the miRs’ downregulation was higher in NG versus both cohorts of pre-diabetics, and in the PDMU versus the PDNMU. The same downregulative trend was observed for inflammatory markers. At the clinical level, both cohorts of pre-diabetics versus NG, and PDNMU versus the PDMU showed a higher rate of MACE at 2 years of follow-up after endarterectomy. Intriguingly, the MACE were predicted by the highest values of miR-24, by highest glucose blood values at hospital admission, and by the diagnosis of atheromatous carotid plaque.

However, the novelty of our study is that metformin therapy could enhance the effects of endarterectomy in pre-diabetics with ACAS by a more significant downregulation of inflammatory molecules and miRs’ expression. Notably, this effect was linked to the significant reduction of MACE at the follow-up end. In this context, we might speculate that the metformin therapy by the amelioration of glucose homeostasis and of the insulin resistance might significantly reduce the inflammatory burden in patients with pre-diabetes [13]. Furthermore, the metformin therapy in addition to endarterectomy might cause, at 2 years of follow-up, the significant downregulation of miRs in ACAS patients with pre-diabetes. Thus, we might speculate that metformin’s pleiotropic effects might consequently reduce the MACE in ACAS patients with pre-diabetes. Conversely, despite the significant miRs’ downregulation seen in any cohort of study after endarterectomy, this effect was inferior in pre-diabetics versus NG, and specifically in PDNMU versus PDMU patients.

In this context, it is well known that the over-inflammation could promote the atherosclerotic processes in ACAS patients with pre-diabetes, leading to plaque instability and rupture [6]. These effects could consequently cause a higher rate of MACE in pre-diabetics versus NG. Intriguingly, we found that the serum expression of miR-24 predicted the MACE at 2 years of follow-up after endarterectomy, with a 3.8 folds’ higher risk to have MACE in the ACAS population. In this context, the miR-24 is implied in complex atherosclerotic processes and angiogenesis [14]. Indeed, miR-24 is a regulator of angiogenesis-related functions via upregulation of matrix metalloproteinase 7 (MMP7), vascular endothelial growth factor receptor 1 (VEGFR1), and hepatocyte growth factor (HGF), and downregulation of vascular endothelial growth factor-A (VEGF-A) [14]. In detail, miR-24 is involved in angiostatin and endostatin production, which are linked to recurrent ischemic cerebral events [14]. However, we might monitor the circulating miR-24 as a promising diagnostic and prognostic serum marker for ACAS patients with pre-diabetes. On other hand, also the miR-27, miR-100, miR-126, and miR-133 are part of a cluster of miRs linked to carotid artery plaque instability, and worse prognosis in ACAS patients [7,8]. Indeed, these miRs are regulators of the inflammatory cascade, and promoters of changing of vascular smooth muscle cells (VSMCs) from the contractile phenotype to the active synthetic phenotype [7,8]. Notably, the VSMCs proliferation and migration from the media to the intima of carotid vessels, could lead to the excessive production of extracellular matrix that could secondary lead to the transformation of atherosclerotic carotid plaque, and then favoring its rupture [7]. Therefore, we might see all these miRs as epigenetic regulators of endothelial progenitors’ cell migration and proliferation in the atherosclerotic cap with consequent plaque instability and rupture in ACAS patients with pre-diabetes [7,8,14]. In this context, the highest glucose blood values at hospital admission predicted the risk to have MACE after endarterectomy at 2 years of follow-up. Indeed, the processes of atherosclerosis could be accelerated in patients with hyperglycemia [4,5]. To date, hyperglycemia could induce pro-atherogenic effects, interfering with arterial remodeling, angiogenesis, and apoptosis by a complex multi-factorial interplay and by the cross-talking with inflammatory, angiogenetic and apoptotic pathways [4]. Therefore, the hyperglycemia might contribute to the plaque instability and rupture, with consequent worse prognosis in ACAS patients. In addition, in our study we found that the patients with pre-diabetes versus NG had higher rate of atheromatous plaques. Notably, the diagnosis of atheromatous plaques increases of 5.4 folds the risk to have Ma ACE at 2 years of follow-up after endarterectomy. This concept has been previously investigated by authors [4]. Indeed, the atheromatous plaques have higher risk of rupture and subsequent thromboembolic/ischemic events and strokes in the overall population, and specifically in patients with pre-diabetes [4,9]. To date, our data taken together have shown an increased risk of progression and rupture of the atherosclerotic carotid plaque in patients with pre-diabetes, and especially in those with worse glycemic control and insulin resistance (non-metformin users). Notably, a recent meta-analysis evidenced that the patients with pre-diabetes had higher rate of atherosclerotic disease and plaques progression of carotid arteries [15,16]. To date, this could contribute to a more severe progression of the cardiovascular disease with an increased risk of recurrent stroke and a worse prognosis [16]. In our opinion, this could be li to the over-expression of miRs, and inflammatory molecules implied in atherogenesis, plaque instability and rupture, as mainly seen in pre-diabetics versus normoglycemics. Then, in this setting, the metformin therapy, by the reduction of glycemia and insulin resistance, could modulate the expression of miRs, and particularly of the miR-24, that could be marked as a predictor of worse prognosis in ACAS’ patients. Therefore, we might suggest the treatment with metformin to reduce the risk of cardiovascular disease progression and of worse prognosis in ACAS patients with pre-diabetes.

This study could present few limitations. We did not perform magnetic resonance imaging exams to study the atherosclerotic plaque stable versus unstable phenotype. Besides, we did not have hemodynamic and biomechanical stress data, and we cannot assess their association with plaque data. Again, the ex vivo model’s loss cannot furnish us conclusive data regarding the metformin-induced effects on inflammation, angiogenesis and apoptosis, and miRs’ expression in atherosclerotic stable/unstable cap of humans. In addition, we did not practice the analysis of inflammatory burden and miRs expression at the level of plaques’ specimens, but this was outside of the scope of the present analysis. Finally, we did not assess other miRs which are also associated with vascular inflammation, such as miR-10a, miR-663, miR-92a, miR-21, and miR-1185 [17], and we did not use high sensitivity C reactive protein, which is the most promising biomarker of preclinical atherosclerosis [18]. Thus, we believe that future prospectively randomized, large-scale clinical trials are required to clarify further the existing relationship between pre-diabetes, inflammatory burden, and circulating miRs in ACAS patients. In addition, further investigations are required to determine whether metformin therapy could significantly reduce the inflammatory burden, miRs’ expression, and MACE in ACAS patients.

## 5. Conclusions

In ACAS patients, pre-diabetes might cause overexpression of inflammatory molecules, and miRs implied in carotid plaque instability, rupture, and MACE at 2 years of follow-up after endarterectomy. In this context, the metformin therapy might ameliorate clinical outcomes after endarterectomy in ACAS patients with pre-diabetes by the reduction of inflammatory burden and by significant down regulation of the miR-24, miR-27, miR-126, and miR-133. Finally, the evaluation of the circulating miR-24 could predict worse prognosis in ACAS patients. To date, the miRs could represent new biomarkers of plaque instability and worse outcomes in ACAS patients with pre-diabetes versus normoglycemics.

## Figures and Tables

**Table 1:** Characteristics of study population at baseline.

Patients Characteristics	NG *n* (125)	PDNMU *n* (73)	PDMU *n* (36)	*p*
Age, years	66.7 ± 6.5	68.8 ± 7.2	69.7 ± 9.2	0.104; 0.130; 0.891
Males, *n* (%)	87 (69.6)	52 (71.2)	25 (69.4)	0.752; 0.562; 0.412
**Patients characteristics**				
Hypertension, *n* (%)	86 (64.8)	61 (83.6)	31 (86.1)	0.05 *; 0.011 **; 0.256
Hypercholesterolemia, *n* (%)	84 (67.2)	58 (79.5)	29 (80.5)	0.001 *; 0.004 **; 0.150
Cigarette smoking, *n* (%)	17 (13.6)	10 (13.6)	5 (13.9)	0.859; 0.870; 0.451
Ischemic heart disease, *n* (%)	45 (36)	35 (47.9)	18 (50)	0.05 *; 0.04 **; 0.120
BMI (kg/m^2^)	26.8 ± 1.9	28.4 ± 1.8	28.0 ± 1.7	0.001 *; 0.001 **; 0.145
Systolic blood pressure (mmHg)	124.7 ± 10.1	129.4 ± 12.8	129.5 ± 9.1	0.018 *; 0.007 **;0.530
Diastolic blood pressure (mmHg)	72.1 ± 6.7	77.7 ± 7.1	76.1 ± 7.9	0.035 *; 0.05 **;0.189
Heart rate	84 ± 8	83 ± 6	84 ± 6	0.175; 0.321; 0.128
HbA1C (%)	5.3 ± 0.4	6.2 ± 0.8	5.8 ± 0.4	0.010 *; 0.022 **; 0.050 ***
Blood glucose (mmol/L)	86.8 ± 15.3	127.8 ± 18.1	105.6 ± 15.5	0.001*; 0.05 **; 0.032 ***
HOMA-IR	4.1 ± 0.25	5.1 ± 0.69	5.0 ± 0.72	0.045*; 0.05 **; 0.05 ***
Total cholesterol, mg/dL	178 ± 32	206 ± 44	187 ± 25	0.001 *; 0.018 **; 0.001 ***
HDL cholesterol, mg/dL	46.8 ± 10.6	40.2 ± 15.8	42.9 ± 12.6	0.005 *; 0.012 **; 0.05 ***
LDL cholesterol, mg/dL	103.9 ± 35.1	128.1 ± 48.4	114.3 ± 27.5	0.035 *; 0.048 **; 0.050 ***
Triglycerides, mg/dL	131.3 ± 23.2	167.9 ± 44.9	134.5 ± 40.7	0.004 *; 0.133; 0.05 ***
Creatinine, mg/dL	1.0 ± 0.1	1.1 ± 0.2	1.0 ± 0.1	0.082; 0.141; 0.520
**Plaque characteristics**				
Stenosis severity, %	75.8 ± 4.1	76.8 ± 4.8	75.9 ± 4.4	0.207; 0.315; 0.135
**Plaque morphology:**				
-Fibrous, *n* (%)	37 (29.6)	16 (21.9)	9 (25)	0.168; 0.171; 0.180
-Fibro atheromatous, *n* (%)	43 (34.4)	21 (28.8)	11 (30.6)	0.208; 0.121; 0.560
-Atheromatous, *n* (%)	45 (36)	36 (49.3)	16 (44.4)	0.05 *; 0.05 **; 0.120
Active therapy (%)				
Aspirin, *n* (%)	107 (85.6)	65 (89)	33 (91.6)	0.120; 0.132; 0.891
Warfarin, *n* (%)	3 (2.4)	2 (2.7)	1 (2.8)	0.252; 0.230; 0.158
β-Blockers, *n* (%)	66 (52.8)	39 (53.4)	19 (52.7)	0.270; 0.260; 0.713
Calcium-channel blockers, *n* (%)	3 (2.4)	4 (5.5)	2 (5.5)	0.05 *; 0.03 **; 0.301
Statins, *n* (%)	112 (89.6)	67 (91.8)	32 (91.6)	0.001 *; 0.002 **; 0.253
ACE inhibitors, *n* (%)	41 (32.8)	36 (49.3)	17 (47.2)	0.001 *; 0.001 **; 0.320
Diuretic agents, *n* (%)	6 (4.8)	8 (10.9)	4 (11.1)	0.05 *; 0.03 **; 0.301
ARBs, *n* (%)	21 (16.8)	21 (28.8)	10 (27.7)	0.001 *; 0.001 **; 0.289

Data are presented as mean ± SD (standard deviation), or number (%). * Is for statistical significant (*p* < 0.05) for normoglycemics (NG) versus pre-diabetics non-metformin users (PDNMU); ** is for statistical significant (*p* < 0.05) for NG versus pre-diabetics metformin users (PDMU); *** is for statistical significant (*p* < 0.05) for PDNMU versus PDMU. ACE = angiotensin-converting enzyme; ARBs: angiotensin receptor blockers; BMI = body mass index; Hb1Ac: glycated haemoglobin; HDL = high-density lipoprotein; HOMA-IR: Homeostatic Model Assessment of Insulin Resistance; IHD = ischemic heart disease; LDL = low-density lipoprotein.

**Table 2:** microRNAs (miRs) expression at baseline and at 2 years of follow-up in normolgycemics (NG) versus pre-diabetics non-metformin-users (PDNMU) versus pre-diabetics metformin-users (PDMU), and major adverse cardiac events (MACE) at 2 years of follow-up in normolgycemics (NG) versus pre-diabetics non-metformin-users (PDNMU) versus pre-diabetics metformin-users (PDMU).

	NG *n* (125)	PDNMU *n* (73)	PDMU *n* (36)	*p* Value
**Baseline miRs’ expression**				
miR-24, A.U.	182.15 ± 57.86	1120,04 ± 206.64	458,97 ± 167.74	<0.05 *^,^**^,^***
miR-27, A.U.	0.91 ± 0.04	2.06 ± 0.21	1.54 ± 0.30	<0.05 *^,^**^,^***
miR-100, A.U.	0.77 ± 0.04	1.76 ± 0.22	0.80 ± 0.12	<0.05 *^,^***
miR-126, A.U.	45.64 ± 23.49	108.65 ± 42.71	134.22 ± 70.58	>0.05
miR-133, A.U.	10.82 ± 1.44	14.96 ± 2.22	9.14 ± 2.16	>0.05
**miRs’ expression at 2 years of follow up**				
miR-24, A.U.	11.21 ± 6.36	98.25 ± 21.08	51.24 ± 17.88	<0.05 *^,^**^,^***
miR-27, A.U.	0.13 ± 0.02	0.95 ± 0.10	0.58 ± 0.09	<0.05 *^,^**^,^***
miR-100, A.U.	0.028 ± 0.0004	0.089 ± 0.006	0.059 ± 0.001	<0.05 *^,^**^,^***
miR-126, A.U.	2.79 ± 1.98	29.55 ± 5.89	10.43 ± 5.84	<0.05 *^,^**^,^***
miR-133, A.U.	0.30 ± 0.06	1.74 ± 0.28	0.94 ± 0.28	<0.05 *^,^**^,^***
**Inflammatory markers at baseline**				
CRP (mmol/L)	0.82 ± 0.31	1.13± 0.49	1.10 ± 0.46	<0.05 *^,^**
IL6 (pg/mL)	3.49 ± 0.38	4.41 ± 0.52	4.36 ± 0.43	<0.05 *^,^**
TNFα (pg/mL)	5.56 ± 0.92	6.96 ± 0.56	6.92 ± 0.54	<0.05 *^,^**
**Inflammatory markers at 2 years of follow up**				
CRP (mmol/L)	0.41 ± 0.10	0.92 ± 0.22	0.73± 0.28	<0.05 *^,^**^,^***
IL6 (pg/mL)	3.10 ± 0.31	4.19 ± 0.22	3.52 ± 0.31	<0.05 *, **,***
TNFα (pg/mL)	4.72 ± 0.71	6.52 ± 0.49	5.54 ± 0.38	<0.05 *^,^**^,^***
**Number of MACE, n (%)**	8 (6.4)	27 (36.9%)	6 (16.6)	<0.05 *^,^**^,^***

A.U. = arbitrary unit; CRP: C reactive protein; IL6: interleukin 6; MACE: major adverse cardiac events; miR = microRNA; NG = normoglycemics; PDNMU: pre-diabetics never metformin users; PDMU = pre-diabetics metformin users; TNFα: tumor necrosis factor alpha. Data are presented as mean ± SD, or number (%). * Is for statistical significant (*p* < 0.05) for NG versus PDNMU; ** is for statistical significant (*p* < 0.05) for NG versus PDMU; *** is for statistical significant (*p* < 0.05) for PDNMU versus PDMU.

**Table 3:** Multivariate Cox Regression analysis to predict major adverse cardiac events (MACE) at 2 years of follow-up.

Risk Factors	HR	CI 95%	*p* Value	HR	CI 95%	*p* Value
Age	0.939	0.876–1.006	0.075	0.970	0.930–1.013	0.169
BMI	0.970	0.926–1.261	0.327	1.070	0.809–1.414	0.637
Hypertension	1.188	0.643–2.196	0.582	0.620	0.176–2.187	0.457
Dyslipidemia	1.707	0.921–3.163	0.089	1.425	0.560–3.629	0.457
Metformin therapy	0.185	0.085–0.401	0.001	0.386	0.053–2.823	0.348
Glucose blood values	1.014	1.009–1.020	0.001	1.020	1.001–1.038	0.029 *
Cholesterol blood values	1.012	1.004–1.019	0.002	1.013	0.999–1.027	0.074
ACEi	1.325	0.650–2.702	0.439	0.583	0.159–2.137	0.416
Statins	0.883	0.450–1.730	0.716	0.501	0.166–1.503	0.217
Pre-diabetes	3.003	1.531–5.869	0.001	1.021	0.688–1.201	0.195
Atheromatous carotid plaque	6.388	2.949–13.838	0.001	5.373	1.251–11.079	0.024 *
IL 6	6.246	3.195–12.210	0.001	1.283	0.020–4.003	0.350
miR-24	5.001	1.781–6.122	0.001	3.842	1.768–19.222	0.011 *

In this table, we reported the univariate and multivariate Cox regression analysis to predict MACE at 2 years of follow-up in patients with asymptomatic carotid artery stenosis (ACSA). Thus, we found that admission glucose values (HR 1.020, CI 95% (1.001–1.038), *p* = 0.029), atheromatous carotid plaque (HR 5.373, CI 95% (1.251–11.079), *p* = 0.024), and miR-24 (HR 3.842, CI 95% (1.768–19.222), *p* = 0.011) predicted MACE at 2 years of follow-up. ACEi: angiotensin-converting enzyme inhibitors; BMI: body mass index; CI: confidence of interval; HR: Hazard ratio; IL6: interleukin 6; miR-24: microRNA 24. * Is for statistical significant (*p* < 0.05).

## Data Availability

Data Availability Statement is available on request.
